# Interplay of α/β-Relaxation Dynamics and the Shape of Ionomer Building Blocks

**DOI:** 10.1038/s41598-018-31368-8

**Published:** 2018-09-07

**Authors:** Bruno R. Matos, Rodolfo Politano, José Fernando Q. Rey, Daniel Hermida-Merino, Ulrich Schade, Ljiljana Puskar, Fabio C. Fonseca

**Affiliations:** 1 Instituto de Pesquisas Energéticas e Nucleares, IPEN-CNEN/SP, São Paulo, 05508000 Brazil; 2 Methoden der Materialentwicklung, Helmholtz-Zentrum für Materialien und Energie GmbH, Berlin, 12489 Germany; 3 Universidade Federal do ABC, UFABC, Santo André, 09219170 Brazil; 4 Netherlands Organisation for Scientific Research (NWO), DUBBLE@ESRF, Grenoble, 38000 France

## Abstract

The relation between the α/β relaxations and the shape of the building blocks of ionomer materials is a key factor for programming an important temperature-dependent property: the memory of shape. However, the morphology of ionomers is indirectly obtained via modeling of small angle X-ray scattering (SAXS) data owing to the hardly accessible image characterization of the nanometric building blocks ‒ micelle-like cylindrical polymeric aggregates (radius ~2–6 nm and length >100 nm). Herein, broadband dielectric spectroscopy (BDS) measurements, free from electrode polarization effects, allowed identifying the time and temperature dependence of the polarization of different length scales of the ionomer matrix, and more importantly, by directly providing the aspect ratio of the radius and length of the polymeric aggregates for each desired temperature. This finding is essential for controlling the shape of ionomer based functional products under several stimuli conditions, thereby advancing remarkable applications, such as four dimensional (4D) printing.

## Introduction

Ionomers are considered shape memory polymers due to the ability of fixing, or programming, two or multi macroscopic shapes on different types of stimuli, such as, temperature and moisture content^1^. Such adaptive materials are envisioned as a revolutionary concept and a massive world trend of production of functional parts^1,2^. Associated with additive manufacturing, the shape memory polymers have the ability of reconfiguring the matrix structure to a temporary desired shape according to the ambient stimulus, adding a new dimension to the final product: time. The advance of shape memory ionomers is valuable for several high-performance applications such as artificial muscle design, electrocatalyst binders, polymer electrolyte fuel cells, and four dimensional (4D) printing^1–5^.

Perfluorinated ionomers (PIs) are a specific family of ionomeric materials that displays outstanding physical properties, such as, thin-film nanoconfinement, exceedingly high ion transport, elevated mechanical resistance, and memory of shape^3,6,7^. All of these properties are known to be linked to the PIs morphology. The PIs morphology has been extensively modeled by SAXS in which the building blocks of their microstructure are assigned to long cylinder like polymeric aggregates (*r* ~ 2.5 nm and *L* > 100 nm), adjacently packed together mainly by temporary ionic crosslinks and a low degree of crystallinity^5,6^. Many efforts have been made to characterize the shape of such polymeric aggregates by image characterization techniques^8,9^. The most promising results have been obtained by transmission electron microscopy (TEM) and atomic force microscopy (AFM)^8,9^. However, due to instability of the sample during TEM measurements and the interaction of the sample with the tip in AFM, most of the available data display artifacts and are not representative of the PI morphology^8,9^. Moreover, by means of such techniques, the *in situ* temperature characterization of the polymeric aggregates is very problematic. This issue is aggravated if the morphology information as a function of relative humidity is required. Such drawbacks have been overcome with the use of small angle X-ray scattering (SAXS) in which the ionomer morphology at different sets of temperature and relative humidity can be indirectly obtained via modeling^9,10^. In addition, usually at the α-transition, the peak associated with the radial correlation of the polymeric aggregates (ionomer peak) disappears, and as such, the mechanism for this thermal-triggered disordering, or loss of correlation of the polymeric aggregates, has not been fully understood or modeled^9,11^. The absence of a solid empirical finding relating the temperature and shape of the polymeric aggregates makes the SAXS modeling arbitrary and speculative. Therefore, this issue requires a modeling-independent characterization of the shape and size of the polymeric aggregates as a function of *T* for understanding the shape memory property and advancing 4D printing, for example.

Broadband dielectric spectroscopy (BDS) is a powerful tool to study the dynamics of the polarization due to ion-hopping within ionomeric matrices in a wide range of length scales^6,12^. However, the dielectric spectrum usually displays electrode polarization contributions that mask the characteristic relaxation frequencies within the ionomer matrix^13^. Specifically, this feature has been a main issue for the characterization of PIs, due to the high ion conductivity, and for more than 40 years the assignment of the dielectric spectrum of PIs is an open problem^6,14,15^. Even more recently, from 2014 up to the present day, the old interpretations of PI dielectric spectra have not thrived over the years^6,15^. From 2014 onwards, more recent findings showed a careful characterization of the dielectric spectrum of solutions and hydrated solid films of a one of the mostly studied PI, Nafion^®^^16^. Dilute Nafion solutions represented a simple system for the characterization of the mechanisms of ion-hopping polarization within the polymeric aggregates^16^. The striking result was that PIs exhibit polyelectrolyte-like polarizations instead of the conventional segmental motion relaxations observed in hydrocarbon ionomers, for example. The α and β relaxations observed in the dielectric spectrum were assigned to the ion-hopping polarization along the longitudinal and radial directions of the polymeric aggregates, respectively^16,17^. By adding salt to Nafion solution a rod-to-coil transition occurred and the size and shape of the polymeric aggregates were estimated considering the position of α and β relaxations^16^. An excellent match was obtained for the dimensions of the polymeric aggregates as estimated by SAXS and BDS^16,17^. This finding opens the investigation of the shape/relaxation relation for solid PI films.

Here, the aspect ratio of the polymeric aggregates was directly obtained at a broad range of temperature for Nafion by BDS. The electrode polarizations at very low frequencies (*f* < 10^−2^ Hz) were eliminated in this work by the use of a 4-probe setup or a two-probe having high specific surface area electrodes. Initially we performed variable temperature SAXS, infrared spectroscopy (FTIR), and dynamic mechanical analysis (DMA) on Nafion in order to determine the morphology of the ionic domains, state of the electrostatic interactions among ionic groups, and the thermal transition within the ionic phase, respectively, at rigorously the same experimental conditions. Posteriorly, the SAXS, FTIR and DMA data are confronted to BDS measurements of Nafion.

**Figure 1:**
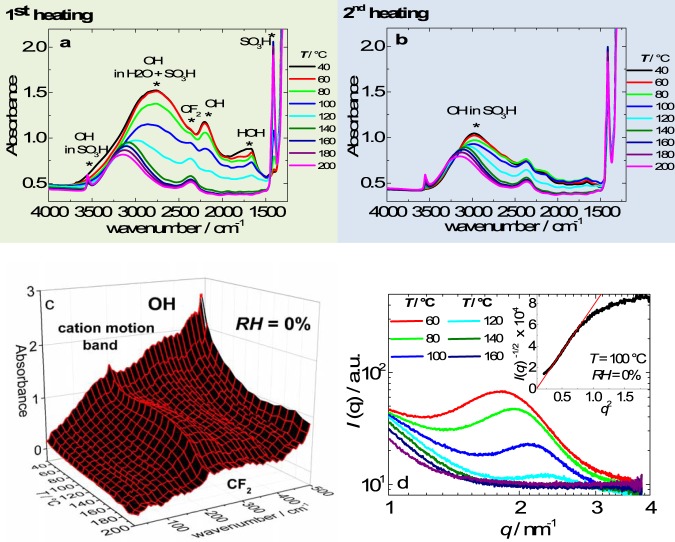
*In situ* MIR temperature measurements of Nafion during the first and second heating in the transmission mode in the functional groups region, 4000–1250 cm^−1^ (**a** and **b**). *In situ* FIR temperature measurements of Nafion during the first heating in the transmission mode using the synchrotron source from the IRIS beamline (**c**). *In situ* SAXS temperature measurements of Nafion during the first heating using the synchrotron source from the ESRF beamline (**d**). The inset shows *I*^−1/2^ vs *q*^2^ plot in the low *q*-range.

Mid and far infrared (MIR and FIR) spectroscopy and SAXS measurements were performed on Nafion during increasing temperature in the 30–200 °C *T*-range (*RH* ~ 0%) under N_2_. Figure 1a and b shows the MIR data in the functional groups region (4000–1250 cm^−1^) collected during the first and second heating, respectively. Figure 1c shows the 2D plot of FIR (400–20 cm^−1^) data for Nafion measured in the first heating. Figure 1d shows the SAXS patterns for Nafion during the first heating, and the inset shows *I*^−1/2^ vs *q*^2^ plot in the low *q*-range.

Relevant features in the Nafion MIR spectra are observed on the first and second heating: (*i)* at 200 °C, the OH stretching at 2980 cm^−1^ displays a blueshift (~220 cm^−1^); followed by the development of a high frequency (3500 cm^−1^) band assigned to dangling OH groups in SO_3_H; (*ii)* the water bands at 1660, 1710 and 2200 cm^−1^ disappeared revealing the underlying CF_2_ overtone at 2370 cm^−1^; and (*iii)* the bands associated with RSO_3_^−^ (1060 cm^−1^, not shown^18^) gradually disappeared leading to the gradual appearance of SO_2_ vibration in RSO_3_H (1410 cm^−1^)^18,19^. Notably, the initial coordination water of Nafion samples results is monohydrated sulfonic groups (RSO_3_H.H_2_O)^20^. Previous thermogravimetric analysis on vacuum dried Nafion samples evidenced that heating from 30–200 °C removed the residual water molecules in the samples^20^. The water removal is in agreement with absence of MIR water bands and the association of the protonic charges with the sulfonic groups at *T* > 100 °C (hereafter mentioned as “dry samples”) indicating that the proton diffusion takes place mostly via ion-hopping. Further minimization of the water content is attained in the second heating (Fig. 1b) in which protons are associated with the sulfonic groups from 40 to 200 °C, due to the presence of the 1410 cm^−1^ band in the entire *T*-range; whilst the broad OH stretching MIR band (3000 cm^−1^) is mostly due to clustering RSO_3_H in the sample^19^. Importantly, the ionic crosslinks persisted in the entire temperature range investigated. The blueshift of the OH stretching in RSO_3_H (2980 cm^−1^) takes place in the α-transition *T*-range (*T*_*α*_ ~ 120 °C), having the onset and endset at 100 and 140 °C, respectively. A blueshift of the OH stretching frequency is reported for solid-liquid-vapor transition of H_2_O and corresponds to the weakening of the hydrogen bonding^21^. The weakening of the OH bonding in Nafion with increasing *T* can be understood considering the counterelastic forces imposed by the main and side chains against the ionic aggregation as the temperature increases. This feature suggests that the enough *KT* energy is being provided allowing segments of the polymer chains to escape from the temporary ionic crosslinks.

Cation exchange experiments in the FIR region reported the cation motion frequency of the H^+^-SO_3_^−^ form in Nafion appearing as broad band centered at 240 cm^−1^, which can also be seen in Fig. 1c ^19,22^. The most prominent features reported in the spectral region of 50–400 cm^−1^ are the CF_2_ rocking band at 204 cm^−1^, and three bands present in the proton form of Nafion∼240, 335, and 375 cm^−1^, which are assigned to H-bond stretching involving the hydronium ion^19,22^. It was previously observed that decrease of hydration promoted a decrease of the intensity of 335 and 375 cm^−1^ bands^19^, following the behavior of the water bands in the MIR range (Fig. 1a and b). However, these bands persisted for *T* > 120 °C, suggesting that these bands are also probably related to OH stretching in RSO_3_H.

It is difficult to infer feature changes of the cation motion band (240 cm^−1^) upon heating due to the partial overlapping with 335 cm^−1^ band (Fig. 1c). Nonetheless, it is possible to notice a broadening and intensity reduction for *T* > 140 °C of the cation motion band. At high *T* ( >180 °C), the absorbances of both 240 and 335 cm^−1^ bands are diminished and only a broad coalesced absorbance is observed. These findings reveal important aspects of the cation-motion band of perfluorinated ionomers. As the cation motion band of ionomers is related to the proton transport across the length scales of the ionic heterogeneities of the sample, the broadening of such band for *T* > 140 °C suggests that the ion-hopping occurs over more disordered ionic aggregates.

Usually, SAXS patterns of Nafion membranes at low *RH* (*RH* ~ 10%) display a scattering maxima centered at *q* ~1.9 nm^−1^ due to the ionomer peak^23^, which can be clearly observed in Fig. 1d for low temperatures. Previously, ultra small angle X-ray scattering (USAXS) measurements of Nafion in the hydrated form displayed low-*q* scattering upturn (*q* < 0.03 nm^−1^), being attributed to large-scale density fluctuations associated with the length of the polymeric aggregates (*l* ≈ 350 nm)^23,24^. Similarly, the Zimm approximation (*I*^−1/2^ vc *q*^2^) is showed in the inset of Fig. 1d to estimate the length of the polymeric aggregates of Nafion in the dry form^23,24^. In the first and second heatings, the length exhibits a weak dependence on temperature. For the first heating, the length decreases from 182 to 174 nm in the 20–200 °C *T*-range, whereas in the second heating the length decreases from 154 to 150 nm at the same *T*-interval. The reduction of the size of the polymeric aggregates, in the sequence 350 nm (hydrated Nafion)^23^ >182 nm (1^st^ heating) > 150 nm (2^nd^ heating), is possibly due to the less expanded polymeric aggregates due to the water elimination and coordination of protons with the sulfonic acid groups, minimizing the electrostatic repulsions among bare sulfonic acid groups in the sample containing water molecules.

The ionomer peak corresponds to the radial correlation of the cylindrical polymeric aggregates, and at 120 °C the radius is 2.74 nm (Fig. 1d). The SAXS patterns of Nafion measured in the first and second heatings are similar in the 30–200 °C *T*-range. With increasing *T*, in the 30–140 °C range, the ionomer peak decreases in intensity, broadens, and displaces to higher *q*-values (from 1.72 to 2.48 nm^−1^). In the second heating, the reduction in SAXS peak intensity in the 30–140 °C range is minimized. Such a less temperature-dependent peak intensity is supported by FTIR (Fig. 1b) and can be related to the loss of residual water molecules and irreversible modification of the ionic network arrangement during the first heating. Most pronounced changes in the SAXS patterns are observed at *T* ~ 140 °C, for which the ionomer peak is totally suppressed. The reduction of the ionomer peak intensity could be linked to the water evaporation. However, MIR results evidenced that the effect of the removal of coordinated water from sulfonic acid groups for *T* > 100 °C are minimal. Therefore, the changes of the ionomer peak features for *T* > 140 °C can be attributed to a loss of ordering of the polymeric aggregates. Such finding is in good agreement with the FIR data, confirming that a structuring of the ionic phase takes place due to the weakening of the electrostatic interactions at the α-transition. Previously, the disappearance of the ionomer peak has been observed for drawn Nafion films in the direction perpendicular to the stretching axis, which was attributed to a misalignment of the polymeric aggregates^24^. In this scenario, a tentative explanation for the suppression of the ionomer peak at the α-transition would be the misalignment of the polymeric aggregates, possibly due to the minimization of the electrostatic repulsion among sulfonic acid groups due to water removal and the weakening of the electrostatic interactions among SO_3_H dipoles, which are the “pillars” for the cylindrical shape of the polymeric aggregates. However, the water removal effect and the weakening of the electrostatic interactions do not explain the reason why the suppression of the ionomer peak takes place at 140 °C. It is also worth noting that the only information that can be extracted from both FIR and SAXS measurements is a disordering of the ionic phase with increasing *T*. Both techniques remain silent with respect to the mechanism of this disordering transition, mechanism of which can be assessed by BDS^25^.

In BDS, the characteristic frequencies of both ion-hopping and α-relaxation are commonly overlapped with electrode polarization at low frequency (typically in the 10^−2^–10^2^ Hz range), hindering the investigation of the dynamics of the dielectric dispersions^17,25^. BDS measurements of Nafion using 4-probe and 2-probe (using high surface specific area electrodes) in the through-plane setup are free from electrode contributions. Such measurements allowed identifying the ion-hopping (proton hopping) as well as the α and β relaxations.

**Figure 2:**
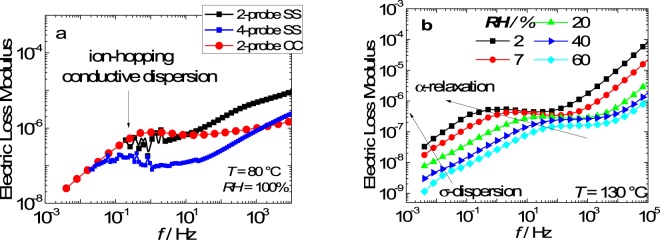
Electric loss modulus of Nafion using three different electrodes setup: 2-probe SS; 4-probe SS; and 2-probe CC (**a**). Electric loss modulus of Nafion with decreasing relative humidity using 2-probe CC (**b**).

Figure 2 shows the dielectric spectroscopy measurements (in the electric modulus formalism/M* = *M*′ + *iM*″) of Nafion performed with decreasing relative humidity from 100% to 2%. Such measurement allows determining the position of the relaxations at high *RH* in which the ion-hopping dispersion (Fig. 2a) and α-relaxation (Fig. 2b) are more easily identified^17^, and monitor its displacement as the sample dries, thereby determining the position of the relaxations in the dry film. The identification of ion-hopping dispersion (σ-dispersion) of Nafion, as showed in Fig. 2a, was performed using three different setups: (*i*) 2-probe using flat stainless steel (SS) electrodes; (*ii*) 4-probe SS electrodes; and (*iii*) 2-probe carbon cloth (CC) electrodes. Both the *M*″(*f*) representation and the 4-probe setup eliminates electrode polarization effects revealing the σ-dispersion at *f* ~ 10^−1^ Hz for Nafion at *RH* = 100%^13^. Such a feature cannot be detected using the 2-probe SS setup. However, highly reproducible DS measurements with a well-defined σ-dispersion are obtained using high specific surface area 2-probe CC electrodes in a broad range of frequency and temperature. The elimination of the electrode polarization in such frequency range allows determining the position of the σ-dispersion and α-relaxation as the relative humidity decreases, as shown in Fig. 2b.

**Figure 3:**
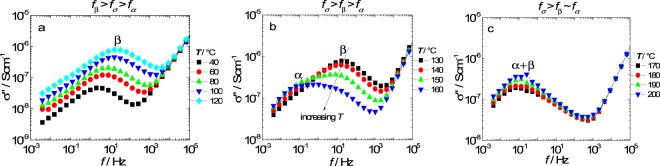
Imaginary component of the proton conductivity as a function of frequency in three temperature ranges: (**a**) from 40 to 120 °C; (**b**) from 130 to 160 °C; and (**c**) from 170 to 200 °C.

At 130 °C (*RH* = 60%) the relaxations are displaced to a higher frequency range in which the σ-dispersion and the α-relaxation are identified at *f*_*σ*_ ~ 10^−1^ and *f*_*α*_ ~ 10^2^ Hz, respectively (Fig. 2b). As the *RH* decreases from 60 to 2%, the α-relaxation decreases in frequency from 10^2^ to 10^−1^ Hz, superposing with σ-dispersion (~ 10^−2^ Hz), indicating a lower *RH*-dependence of *f*_*σ*_ compared to *f*_*α*_. The σ-dispersion frequency of Nafion at dry conditions is in good agreement with previous investigations of *ac* conductive dispersion (*f* ~ 10^−2^–10^−3^ Hz)^17^. Therefore, the results of Fig. 2b indicate that at dry conditions (*T* = 30 °C and *RH* ~ 0%) initial position of the α and β relaxations with respect to the σ-dispersion are: *f*_*σ*_ ~ *f*_*α*_ and *f*_*σ*_ < *f*_*β*_. Such relations are helpful to understand the dynamics role of the ion-hopping on the *T*-dependence of the relaxation dynamics of the ionomer, as shown in Fig. 3.

Figure 3 shows the imaginary conductivity spectra (σ* = *σ*′ + *iσ*′) of Nafion at three *T*-ranges at *RH* ~ 0% during the second heating. In Fig. 3a, only the β-relaxation can be observed in the 40–120 °C *T*-range, indicating that at *RH* ~ 0%, the alpha-relaxation is displaced even further to lower frequencies.

The ion-hopping characteristic frequency, *f*_*σ*_ refers to the dynamics of the temporary crosslinks^25^, while α- and β-relaxations represent the ion-hopping polarization due to the higher activation energy across the length scales of the polymeric aggregates^13^. The protonic charges diffuse via ion-hopping in the polymeric matrix and polarize at the polymeric aggregates. In this context, two limiting cases are relevant for understanding of Nafion spectra: (*i*) for *f*_*β*_ > *f*_*σ*_ > *f*_*α*_, the proton diffusion is not limited by the longitudinal polarization of the polymeric aggregates; and (*ii*) for *f*_*β*_ > *f*_*α*_ > *f*_*σ*_, the proton diffusion is reduced by the polarization of the protonic charges across the radius and length of the polymeric aggregates. The change from behavior (*i*) to (*ii*) takes place at 120 °C, as shown in Fig. 3c and d, indicating that the α-transition of Nafion is associated with the presence of an additional polarization of charges existing at *T* > 120 °C.

Figure 3b shows that as the temperature increases from 40 to 120 °C, the β-relaxation displaces to higher *f* and a shoulder is seen at 120 °C due to α-relaxation high-*f* displacement. In the 140–170 °C *T*-interval (Fig. 3c), the β-relaxation displaces to low *f* until it superposed with the α-relaxation at *f* = 10^-1^ Hz. Both the high-*f* and low-*f* displacements of α and β-relaxations, respectively, represent a change of conformation of the polymeric aggregates. Therefore, the blueshift of α-relaxation indicates a reduction of the length of the polymeric aggregates, whereas the redshift of β-relaxation indicates a thickening of the polymeric aggregates. The coincidence of both relaxations for *T* > 170 °C suggests a reduction of the aspect ratio of the polymeric aggregates to a nearly spherical shape.

The length and radius of the polymeric aggregates (*L*) are associated with the radial (*f*_*β*_) and longitudinal (*f*_*α*_) polarization frequencies by the following equation^12,25^:

1 $${{f}_{\alpha ,\beta }}^{-1}\approx \frac{{L}^{2}}{D}\,,$$

where *D* is the diffusion coefficient of protons counterions obtained by Nernst-Einstein relation: *D* = $\sigma {k}_{b}T/n{e}^{2}$); where *k*_*b*_ is the Boltzmann constant, *T* is the absolute temperature, *e* is the elementary charge, and *n* is the charge concentration. In the *f*_*β*_ > *f*_*σ*_ > *f*_*α*_ regime, at 120 °C, the radius of the polymeric aggregates is~6.47 nm. Due to the difficulty in assessing the α-relaxation from 30 to 120 °C it is not possible to precisely obtain the length of the polymeric aggregates. However, the high-*f* shift of the α-relaxation in Fig. 2b (*f* ~ 10^−2^ to 10^−1^ Hz) suggests a reduction of the length from 287 to 90 nm, in which the latter is the lowest length achieved by the aggregates at *T* = 160 °C (the endset of α-transition). In the 170–200 °C range, the α-relaxation frequency is nearly constant resulting in an increase of the length of the polymeric aggregates ranges from~141 to 210 nm, which can be an outcome of the thermal expansion of the aggregates. It is worth emphasizing that the radius and length obtained by BDS have similar magnitude as the ones obtained by SAXS. The FIR, SAXS and BDS are in very good agreement indicating that the properties of Nafion membranes are profoundly modified above the α-transition due to the crossover from the two regimes and the suppression of the radial correlation of the polymeric aggregates.

**Figure 4:**
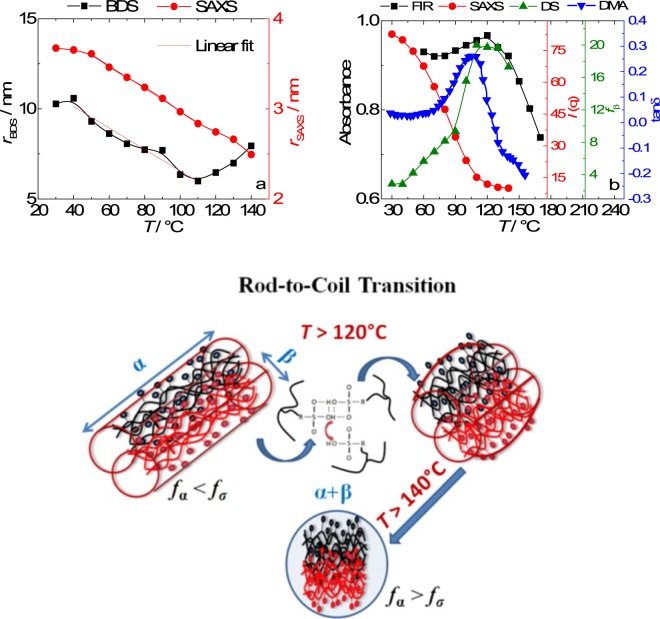
The α-transition as measured by DMA and the disordering of ionic domains of the polymeric aggregates as determined by different techniques, FIR (band position), SAXS (ionomer peak position) and BDS (β-relaxation position) (**a**); the radius of the polymeric aggregates as estimated by SAXS and BDS (**b**); and schematic representation of the proposed conformation transition of Nafion due to the crossover from regime *i* to *ii* (**c**).

Figure 4a shows the radial correlation of the polymeric aggregates (*r*) estimated by SAXS and DS. Figure 4b combines FIR, SAXS and BDS data evidencing the suppression of the radial correlation length at the α-transition, as determined by dynamic mechanical analysis (DMA). Figure 4c shows a schematic representation of the proposed conformation transition of Nafion due to the crossover from regime *i* to *ii*.

In Fig. 4a, as the temperature increases from 30 to 120 °C, the radius obtained by SAXS and BDS of the polymeric aggregates decreases, following a similar power law with increasing *T*, −0.02 and −0.05, respectively.

The α-transition as determined by DMA, the elastic modulus drops to values close to zero confirming that the long-range motion of the main chains is no longer restricted by the ionic crosslinks. Since the ion-hopping is correlated with the motion of the main and side chains, the α- and β-relaxations are associated with the long and short range motions of the polymer backbone, respectively. Similarly, considering the segmental motion of the main chains, the two limiting cases can be devised as: (*i*) For *f*_*β*_ > *f*_*σ*_ > *f*_*α*_, the long range motion of the main chains is restricted by the ionic crosslinks; and (*ii*) for *f*_*β*_ > *f*_*α*_ > *f*_*σ*_, only the short range motions are restricted by the ionic clustering. This conformation transition is only possible due to the *f*_*β*_ > *f*_*α*_ > *f*_*σ*_ relation at *T* > 120 °C, which allowed the long range motion to escape from the restrictions imposed by the ionic crosslinks.

The combined techniques showed that the length scales of the polarizations probed by BDS are compatible with the ones observed by SAXS. Such findings allow correlating conformation changes of the polymeric aggregates with the ion-hopping. Therefore, the understanding of the evolution of the size and ordering of the polymeric aggregates and the ion-hopping with increasing *T* would help establish the relationship between the ionic network and microstructure of PIs, which is crucial for tailoring new high-performance ionomers^26^. The reversibility of the shape of the polymeric aggregates due to annealing and water swelling of Nafion is the next step of this investigation.

## Conclusions

Broadband dielectric spectroscopy (BDS) with free electrode polarization effects is shown to be a unique tool capable of revealing the origin of the atypical dynamics of α-transition in perfluorinated ionomers. The combination of variable temperature IR, SAXS, and DS allowed for monitoring of the electrostatic interactions and morphology from different length scales in Nafion membranes. These characterizations provide further insights into the origin of α-transition. Namely, the α-transition is a critical temperature (*T* = 120 °C) separating two regimes. Below α-transition (*T* < 120 °C) the shape of the polymeric aggregates are locked into nanocylinders due to the fast ion-hopping, which restricts the translational motion of the main and side chains. Above α-transition (*T* > 120 °C), the relaxation frequency of α surpasses the ion-hopping permitting the long-range motion of the main chains, which relaxes in a more spherical conformation. Such finding allows mastering the temperature-shape relationship in ion-containing polymers as well as tuning the desired shape of the building blocks of the ionomer morphology.

## Methods

Commercial Nafion membranes (*EW* = 1,100 g eq^−1^) were obtained from DuPont with different thicknesses (N211, N115). The membranes were pre-treated by standard cleaning and activation protocols^17^. The obtained film was then post-treated in 3% (w/w) H_2_O_2_ and 0.5 M H_2_SO_4_, with intermediate steps in H_2_O to remove excess chemicals. Nafion membranes in the non-ionic form (RSO_2_F) were used to help identifying the IR bands. In all measurements, the water content in commercial Nafion samples (*EW* = 1100 meqg^−1^) was minimized by keeping them in N_2_ for 24 h, which also allowed us to better estimate the initial hydration condition of the membrane^20^.

Variable temperature mid infrared (MIR FT) and far infrared (FIR FT) spectra were measured at the IRIS beamline at the electron storage ring BESSY II of Helmholtz Zentrum Berlin. MIR FT was measured in transmission mode in the Bruker Vertex 70/v using a KBr beamsplitter, a DLaTGS detector fitted with an internal global source. For these measurements a modified Harrick demountable FT IR transmission cell was employed which allowed in-vacuum heating. The cell was equipped with ZnSe windows of 2 mm thickness and a 630 µm PTFE spacer. The spectra were acquired with 4 cm^−1^ resolution and 32 scans were averaged. The references were recorded through the empty channel inside the spectrometer sample compartment under vacuum. FIR FT measurements in spectral region between 600 and 30 cm^−1^ were performed using the Harrick cell equipped with 2 mm thick Ge windows, with 2 cm^−1^ resolution using infrared synchrotron radiation as a source together with a liquid helium cooled silicon bolometer and a silicon beamsplitter. The reference was obtained by measuring the empty Harrick cell with identical conditions in the entire range of temperature investigated. Previous to the measurements, the membranes were preconditioned in the Harrick cell by purging N_2_ (*RH* ~ 0.1%) for 24 h at room *T*. The relative humidity was recorded using a humidity sensor (Sensiriont EK-H4) at the outlet of the cell. The data collection was performed on N211 and N115 samples in the 30–200 °C *T*-range with a step of 10 degrees in two successive heatings. The FTIR bands in the 1100–1200 cm^−1^ range are saturating (not shown). Measurements were performed after ~10 min at each temperature for system stabilization, when no noticeable spectral changes were observed.

The samples were examined by SAXS at the beam line BM26-B^27,28^ at the European Synchrotron Radiation Facility (ESRF) in Grenoble, France. The energy of X-ray source was 12 keV (λ eV0.1033 nm), and sample to detector distance was 3005 mm. Data was recorded using the Pilatus 1 M detector, with 172 × 172 μm pixel size. The samples were heated at 2 °C/min from 20 °C to 250 °C, cooled at 40 °C/min to 20 °C, heated again at 2 °C/min to 300 °C and finally cooled at 10 °C/min to 20 °C, in a Linkam DSC600 hot stage for the simulatenous SAXS temperature-dependent study. The SAXS patterns were reduced by BUBBLE and by the homemade XRTools software. The scattering Intensity (I) *vs* scattering vector, *q* sc4π sin θλ^−1^, where λ is the X-ray wavelength and 2θ is the scattering angle. The scattering vector was calibrated using silver behenate. The scattering patterns were also corrected for transmission, normalized upon primary beam fluctuations and background scattering before data integration.

Dynamic mechanical analysis (DMA) was carried out under nitrogen flow in a Netzsch DMA 242 E in tensile mode. Rectangular Nafion samples (15 × 6 mm^2^) were cut and tested, respecting the machine direction. The measurements were performed from 30–200 °C with 10 °C steps under N2 flowand amplitude of 4 μm and oscillation frequency of 1 Hz.

Broadband dielectric spectroscopy (BDS) data were collected in a specially designed air-tight sample holder able to measure the proton conductivity in the range *T* = 30–200 °C with *RH* = 0% (dry N_2_ purge)^17^. Temperature controllers connected to band heaters placed externally around the cylindrical chambers are monitored by thermocouples (type K) inserted inside the metallic walls. The sample holder is capable of controlling both the temperature (from room temperature up to ~200 °C) and the relative humidity (*RH*, from ~3 to 100%). Nafion samples were sandwiched between stainless steel spring-load contact terminals (electrically insulated from the chamber walls) with carbon cloth to facilitate water equilibration. In this experimental apparatus, the *RH* of the sample chamber can be calculated by, *RH* = ρ(*T*_r_)/*P*(*T*_c_) × 100, where ρ is the vapor partial pressure, *P* is the saturated vapor partial pressure, and *T*_r_ and *T*_c_ are, respectively, the water reservoir and sample chamber temperatures. A Solartron 1260 frequency response analyzer was used in the frequency (*f*) range of 4 mHz to 3 MHz applying an *ac* amplitude of 100 mV. The complex conductivity (σ* = 2*πfε*_0_ε*) and electric modulus (M* = *M*’ + *iM*” = 1/ε*) representations were used throughout this study, in which the dielectric loss (*ε′*(*f*)) was obtained from:

2 $$\varepsilon ^{\prime} (f)=\,-\,\frac{dcos(\theta (f))}{2\pi f{\varepsilon }_{0}S|Z(f)|},$$

where *ε*′ and *ε*′ are the real and imaginary parts of the dielectric permittivity; *ε*_0_ is the vacuum permittivity (~8.854 × 10^−14^ Fcm^−1^); *S* is the electrode active area, *d* is the thickness of the membrane; |*Z*| and *θ* are the modulus and phase angle of impedance. The frequency dependent conductivity was obtained using the relation: *σ*′ = 2π*fε*_0_*ε*′^13^.
